# Mortality of the invasive white garden snail *Theba pisana* exposed to three US isolates of *Phasmarhabditis* spp (*P*. *hermaphrodita*, *P*. *californica*, and *P*. *papillosa*)

**DOI:** 10.1371/journal.pone.0228244

**Published:** 2020-01-29

**Authors:** Irma Tandingan De Ley, Jacob Schurkman, Cheryl Wilen, Adler R. Dillman

**Affiliations:** 1 Department of Nematology, University of California, Riverside, California, United States of America; 2 University of California Cooperative Extension, San Diego, California, United States of America; UMASS Medical School, UNITED STATES

## Abstract

*Theba pisana* is a serious snail pest in many parts of the world and affects diverse crops including grain, vegetables, grapevines, and ornamental plants and shrubs. Due to its gregarious nature, ability to reproduce rapidly, and the difficulty of controlling it by conventional methods, it has the potential to become a significant pest where introduced. Mitigating this pest is an important challenge that must be addressed. *Phasmarhabditis hermaphrodita*, is a gastropod-killing nematode that is commercially available only in Europe (Nemaslug ®) and Sub-Saharan Africa (Slugtech ® SP). The use of effective gastropod-killing nematodes in the genus *Phasmarhabditis* (*P*. *hermaphrodita*, *P*. *californica* and *P*. *papillosa*) in California may provide one strategy for alleviating the potential damage and further spread of these snails, which are currently limited to San Diego and Los Angeles counties. Laboratory assays demonstrated for the first time that US isolates of *P*. *hermaphrodita*, *P*. *californica* and *P*. *papillosa* at 150 DJs/cm^2^ caused significant mortality and are equally lethal to *T*. *pisana*. Molluscicidal efficacy of these nematodes are comparable with those of iron phosphate, at the recommended high dose of 4.88 kg/m^2^. Additional trials are needed to determine their effects at lower dose and whether they are dependent on the size or age of the snails.

## Introduction

Terrestrial gastropods are land-dwelling snails and slugs in the class Gastropoda (Phylum: Mollusca). Most terrestrial gastropods consume both live and dead plant matter, serving a vital role in natural ecosystems by breaking down these plant materials and fertilizing the soil. However, invasive gastropods that are introduced to an ecosystem in which they thrive can be detrimental as they can negatively affect agricultural and commercial crops [1, 2]. Invasive gastropods are significant pests of agriculture and horticulture in North America with many species feeding directly on and reducing the yield and quality ratings of a wide range of crops [3–5]. Some species can vector human pathogens such as the potentially fatal *Angiostrongylus cantonensis* (Chen, 1935) [6]; and also threaten populations of local organisms resulting in severe biodiversity loss [7].

The white garden snail or Italian white snail *Theba pisana* (Müller, 1774) has been transported out of its own native range multiple times across the globe and been introduced in many countries including Australia, the United States, South Africa, and others [8]. Its native range is considered to be along the Mediterranean and much of Western Europe [8, 9], and it has established itself as an important pest in most of the regions it has invaded [9, 10].

*T*. *pisana* was first detected in North America in La Jolla, San Diego county in 1914 [11, 12] and was considered one of the worst snail pests of agriculture and horticulture ever introduced into the continent at that time [13]. Its presence was later reported in Los Angeles and Orange counties [14] but considered eradicated in 1972 only to reappear in August 1985 in a 10-square mile area in San Diego county [15]. Although it is found throughout San Diego county, it is officially established only in certain coastal and inland areas in San Diego county, a coastal site in Los Angeles county [16], and has also been detected in Orange county though it is not considered established there [17].

It is a B-rated pest in California, i.e., it is “an organism of known economic importance subject to: Eradication, containment, control or other holding action at the discretion of the individual county agricultural commissioner; or an organism of known economic importance subject to state endorsed holding action and eradication only when found in a nursery” (https://ucanr.edu/sites/plantpest/Regualtory_Information/Pest_Ratings/).

**Fig 1:**
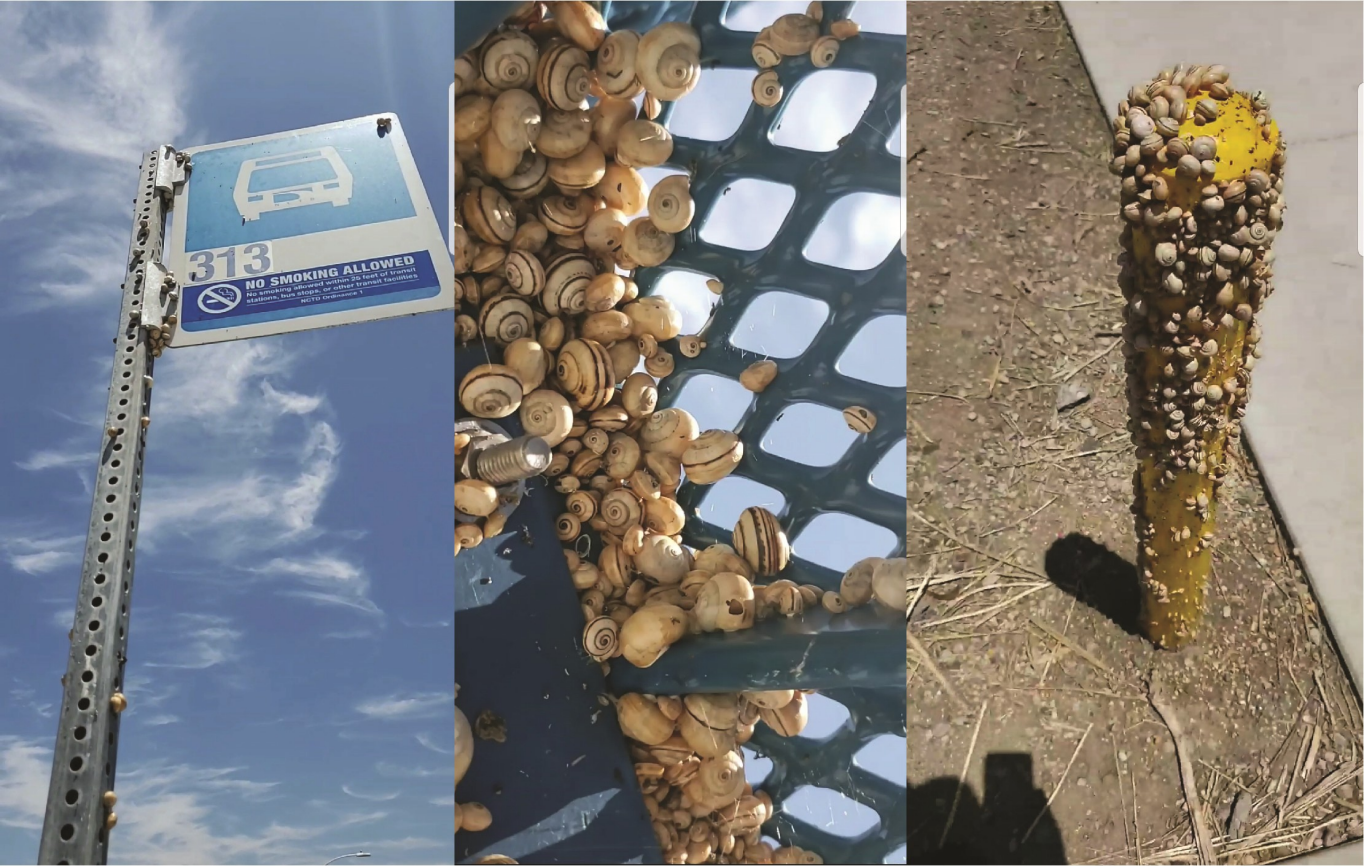
Left to right: *Theba pisana* aestivating on a bus stop sign; beneath a bus stop steel bench and on a yellow pole near a sidewalk in Oceanside, CA.

The snails often grow to extremely large populations and crawl up plants with large stalks where they aestivate for hot and dry periods of the year [9, 10]. With high populations, *T*. *pisana* can also aestivate on man-made structures where they have become a nuisance (Fig 1); and are known to reach densities of up to 3000 snails per tree in California, which along with a rapid rate of reproduction, are primary factors that can make this snail a major pest [13–15]. The snails become more active during the wet seasons and feed on the leaves and stems of the plants. They have caused significant damage to ornamental flowers, vegetables, citrus, almond, olive trees and grapevines in Australia [10, 18–20]. Additionally, the snails have been found to damage equipment and livestock. Due to the snail’s aestivation in large clumps on plants, the machinery that harvests these plants in agricultural operations may get clogged and break down. Livestock have been found to reject pasture and hay heavily contaminated with *T*. *pisana* [9, 20–22]. In California, the snails have been observed feeding on fruit trees and ornamental nursery crops. Massive numbers of aestivating *T*. *pisana* are regularly found on trees and shrubs, reducing the aesthetic value of ornamental landscaping (Wilen, per. obs.). Nursery shipments have been rejected where the snail has been detected.

Mitigating damage due to this invasive species usually requires a combination of methods that include the use of metaldehyde baits; detailed surveys of the ground, plants, and structures within the infested areas [15]; sprayable molluscicides, burning; hand picking [12, 14, 23]; and the use of traps and barriers [24]. Chemical molluscicides are effective and can cause significant mortality against populations of *T*. *pisana* [25, 26]. However, they are non-selective and may be toxic to beneficial and native mollusks [14] and non-target organisms like earthworms and fishes. The use of molluscicides also risks groundwater contamination [27] and continuous use may lead to gastropod tolerance [28].

Biological control lack these issues and could offer a safer, environmentally-friendly alternative. Extensive work has been done on biological control agents for invasive gastropods. One successful example is the nematode *Phasmarhabditis hermaphrodita* (Schneider, 1859) Andrássy, 1983. It has been marketed commercially (Nemaslug®) for application on vegetables, high-value crops and field crops in the UK, Ireland, France, The Netherlands, Belgium, Germany, Denmark, Norway, Finland, Poland, Spain, the Czech Republic, Italy, and Switzerland [29]. It has been successfully used to control gastropods in commercial settings such as nurseries, home gardens, greenhouses and field crops [30–32], often with efficacy comparable to and sometimes better than molluscicides. The nematode is able to parasitize a wide range of gastropod species with slugs being their main susceptible hosts. *P*. *hermaphtrotida* is know to infect at least 17 slug species belonging to families Limacidae, Ariolimacidae, Arionidae, Milacidae, Testacellidae, Vagnulidae; and 8 snails from Acatinidae, Helicidae, Cochlidellidae, Hygromiidae, and Lymnaeidae, [29, 33] (Tandingan McDonnell, GALS). However, despite its recorded success in Europe, this product has not been marketed in the US, because it was considered an exotic species. However, multiple species of *Phasmarhabditis* (*P*. *californica* Tandingan De Ley, Holovachov, Mc Donnell, Bert, Paine, & De Ley, 2016; *P*. *hermaphrodita*, and *P*. *papillosa* (Schneider, 1866) Andrássy, 1983) have been recovered in California, USA [34, 35] and Oregon [36]. This discovery has opened possibilities for research toward a better understanding of the biological control potential of these locally-isolated species in managing pest gastropods, either alone or as a component of integrated pest management (IPM).

Among these species, *P*. *hermaphrodita* was reported to successfully control *T*. *pisana*, where significant mortality was observed both in the lab and in the field within 4–21 days post application [37, 29]. While the efficacy of *P*. *hermaphrodita* against *T*. *pisana* has been tested, the potential of other *Phasmarhabditis* species, such as those that were recovered in California have not been determined. This paper is the first report on the comparative efficacy of three *Phasmarhabditis* species isolated in the US against the emerging pest, *T*. *pisana* in California.

## Materials and methods

### Arena design

**Fig 2:**
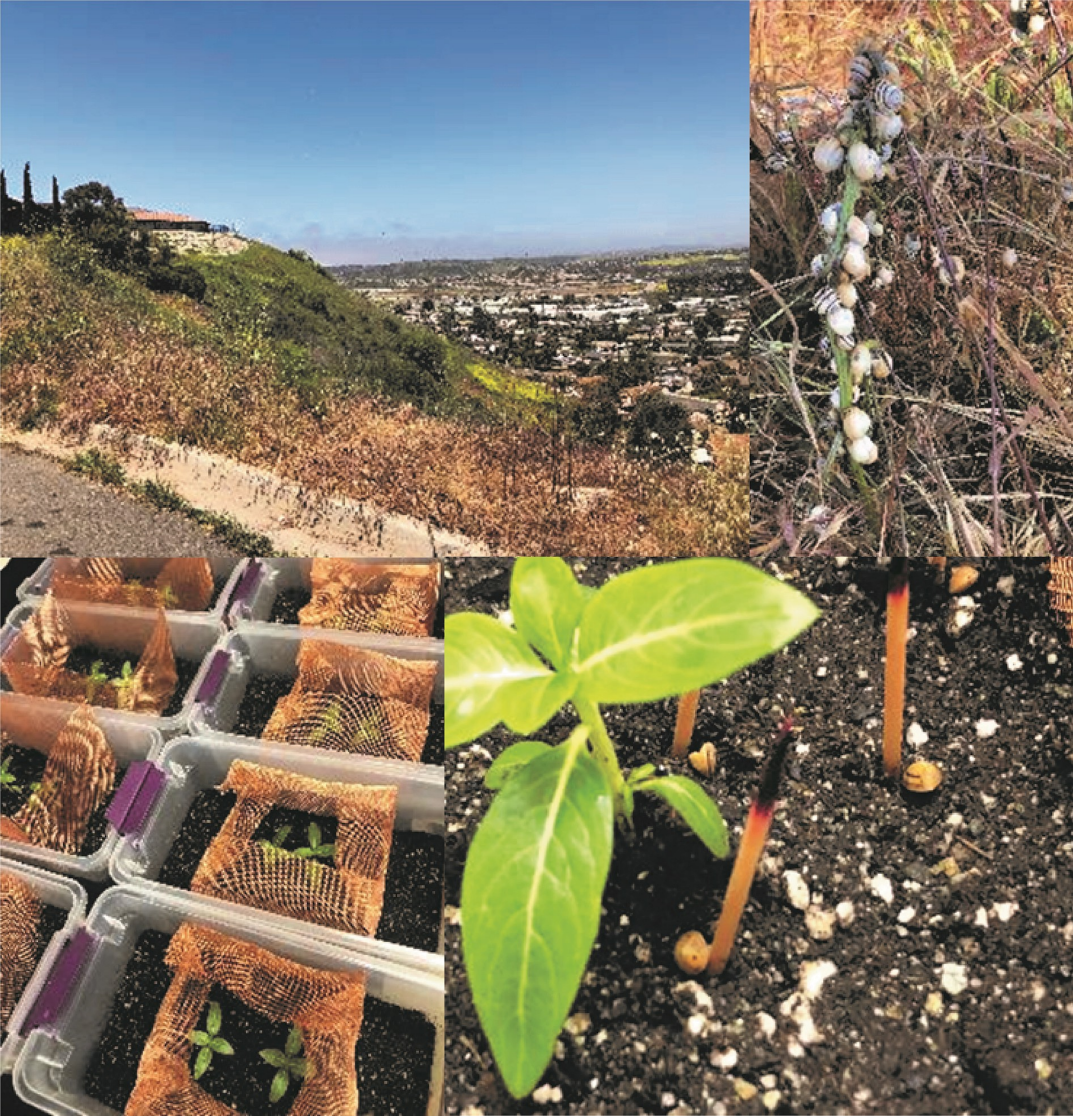
Clockwise from top left: The collection site of *Theba pisana* in a vacant lot in Oceanside, San Diego (33° 12’35” N 117° 20’30”W); snails on the vegetation; treatment arenas; and dead snails 7 days after treatment.

The test arenas consisted of a tray (33.5cm L x 11.5cm H x 18.5cm W) filled with three layers of (a) pea gravel (350mL) at the bottom, (b) a fabric barrier (Dewitt 3' x 100' 6 Year Weed-Barrier Landscape Fabric) that fitted the tray and (c) 600g of autoclaved soil (75% SunGro Sunshine No. 4 mix and 25% UC soil mix 3 [38]). Soil moisture was adjusted by adding 600 mL of deionized water to each arena. Two six-week-old periwinkle (*Vinca minor* L.) were planted 3 cm to the left and right of the arena’s center. The movement of *T*. *pisana* was tested for a week when the snails were presented with copper wire as a barrier along the edges of the arena. After testing, it was noted that the snails were thwarted by the material and were aestivating on the V. minor plants rather than the walls of the container or the copper wiring. Therefore a 16.5 x 16.5cm2 area in the middle of the arena was enclosed with the copper wire to prevent snails from escaping the container, as well as to provide a place for aestivation (Fig 2).

### Nematode preparation

The nematode inocula were prepared from modified White traps [39] on freshly killed *Ambigolimax valentianus* Férussac, 1822 inoculated with mixed stages of each species isolate and incubated at 17°C. The slugs were inoculated with xenic cultures of *P*. *californica* (ITD726), *P*. *hermaphrodita* (ITD272) and *P*. *papillosa* (ITD510). Only dauer stages were used by collecting the three-week-old extracts into individual tissue culture flasks. The dauers were quantified and standardized prior to use. This was performed by counting the number of dauer juveniles (DJs)/10 μL and using the average of five counts, the required volume/arena was determined, pipetted into individual conical tubes and the final volume adjusted to 10 mL using double-distilled water.

We only used five times (150 DJs /cm^2^) the recommended rate of Nemaslug® (30 DJs/cm^2^) for *P*. *californica*, *P*. *hermaphrodita* and *P*. *papillosa* due to space limitation in the growth chamber and this being our first three-species comparison using local isolates on *T*. *pisana*. We also used the higher recommended dose of 4.88 kg/m^2^ of iron phosphate (Sluggo Plus®, active ingredients (a.i.) are: 0.97% iron phosphate and 0.07% Spinosad (a mixture of spinosyn A and spinosyn D)) as molluscicide control; and provided no nematode, snail-only treatment for comparison. We specifically chose iron phosphate rather than metaldehyde because the use of metaldehyde is not considered organic and we aimed to compare control methods that are compatible with organic practices.

### Experimental set-up

Areas in San Diego county, California that are known to harbour high levels of *T*. *pisana* infestation had been previously surveyed and identified by CW as the Area IPM Advisor. Snails were collected from a vacant lot adjacent to residential areas in Oceanside, California (33^O^ 12’35” N 117^O^ 20’30”W), (Fig 2) under CDFA permit 3449. For this experiment, we used small snails ranging from 0.25–1 gram each, measuring 4–6 mm at the widest shell diameter using a ruler.

Ten pre-weighed snails of this size range were introduced on the soil around previously-planted two six-week old *V*. *minor*. Immediately after snail introduction, the nematode inocula were slowly and evenly applied, using a spray bottle on the soil. The number of dead snails was recorded daily for seven days. Dead snails are usually upside down and immobile, but to further confirm this, a toothpick was used to prod the snail to move, and the toothpick was used to mark the snail’s location by placing next to the snail. If the snail had not moved the following day, the toothpick was colored purple and the snail was then confirmed dead the previous day (Fig 2). In addition, dead snails usually had either withdrawn foot muscles and without the thin layer of dried mucus or epiphragm which are typical for estivating snails. Treatment arenas were replicated three times, repeated twice, and maintained in a diurnal growth chamber with alternating temperatures of 20°C and 15°C and 12-hour daylight.

All statistical analyses were performed with GraphPad Prism 8.2.1. The snail mortality data was tested for normal (Gaussian) distribution and log normal distribution. To test for this, we used the Anderson-Darling test, the D’Agostino-Pearson omnibus normality test, the Shapiro-Wilk normality test, and the Kolmogorov-Smirnov normality test with Dallal-Wilkinson-Lilliefor’s test for P value. Each treatment failed the normality and log-normality test with the exception of the treatment with iron phosphate which passed the normality test using the Kolmogorov-Smirnov test (P = 0.0732). Due to the non-normal distribution of data, we used non-parametric methods to analyze the data. Analyses were performed using a Kruskal-Wallis test; and a two-stage linear step up procedure of Benjamini, Krieger, and Yekutieli for the multiple comparisons test comparing the mean rank of each day (days after exposure or DAE) and treatment (*Phasmarhabditis* spp and iron phosphate) combination with every other day and treatment combination. All data are included as a supplemental data sheet.

## Results

**Fig 3:**
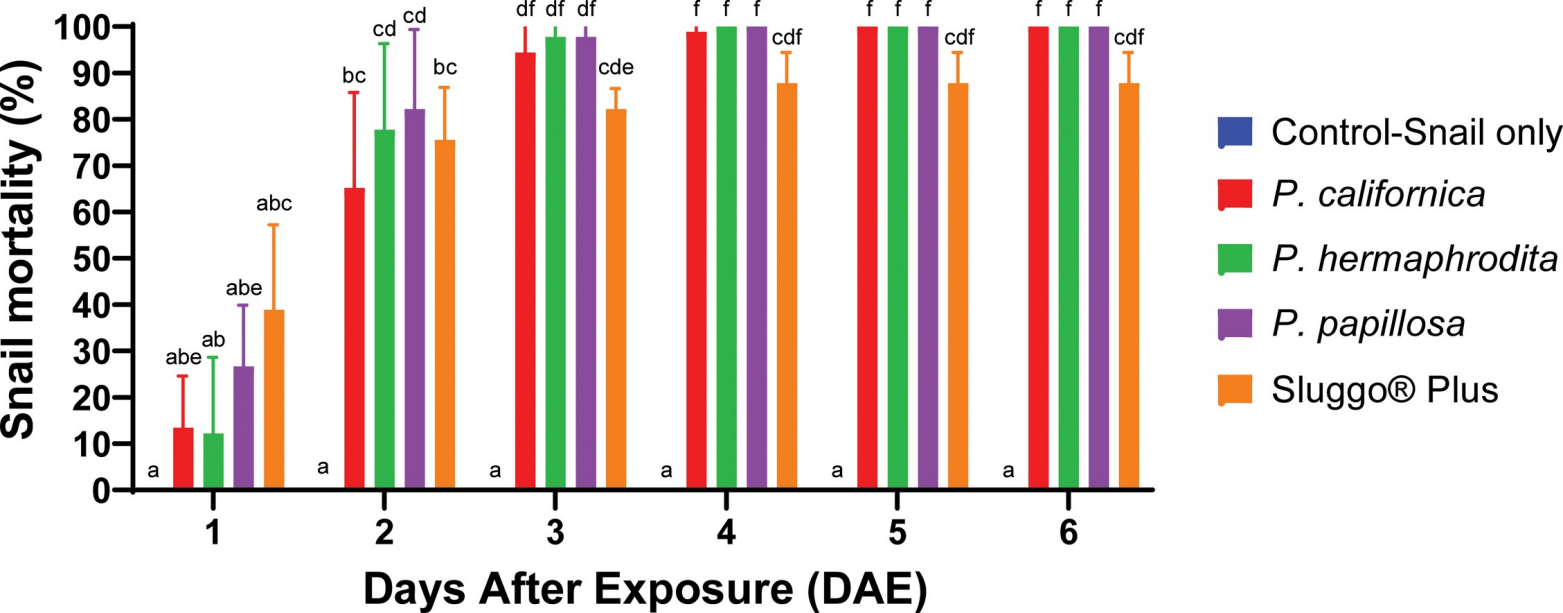
Mean (±SD) percent mortality of *Theba pisana* 1, 2, 3, 4, 5, and 6 days after exposure to 5 times the recommended rate (150 dauer juveniles/cm^2^) of US strains of *Phasmarhabditis californica*, *P*. *hermaphrodita*, *P*. *papillosa*, and iron phosphate (Sluggo Plus®) in a closed container filled with moistened soil and two *Vinca minor* plants. Control was a snail only treatment. Bars with different superscript letters denote a significant difference between the means using a Kruskal-Wallis test; and a two-stage linear step up procedure of Benjamini, Krieger, and Yekutieli for the multiple comparisons test.

*T*. *pisana* began to die 1 DAE, however, mortality was insignificant compared to the non-treated control (Fig 3). All treatments caused significantly higher mortality compared to the non-treated control by 2 DAE (*P*. *hermaphrodita*, P = 0.0033; *P*. *californica*, P = 0.0315; *P*. *papillosa*, P = 0.0013; iron phosphate, P = 0.0154). Mean mortality significantly increased with increasing time of exposure, with comparable means of 65.2% (SD = 20.6, N = 9) to 82.2% (SD = 17.2, N = 9) for all nematode and iron phosphate treatments. At 4 DAE, lethal effects of all treatments were not significantly different from each other, with *P*. *papillosa* and *P*. *hermaphrodita* causing 100% mortality; *P*. *californica*, 98.9% (SD = 3.3, N = 9); and iron phosphate, 87.8% (SD = 6.7, N = 9). There was no mortality on the non-treated snails from 1–6 DAE. These results suggest that all three *Phasmarhabditis* species at 150 DJs /cm^2^ and iron phosphate at the higher dose of 4.88 kg/m^2^ are equally effective at killing *T*. *pisana*.

There was hardly any damage on the *Vinca* plants even at six days after the experiment commenced, or the day it was terminated. Non-treated plants had very few tiny specks or holes on leaf surfaces and live snails from control treatments were aestivating on the plant leaves and stems. Therefore it was not possible to determine feeding inhibition due to *Phasmarhabditis* exposure. There were no obvious symptoms of disease progression as the healthy snails are either mostly stuck to the plant, very few on the soil, and are inverted when dead. Upon examination of the dead snails, the nematodes were actively moving outside and inside the shell, the opening completely covered with various stages of the nematode, with mostly gravid females and juveniles at1st and 2nd stages; and snail tissues completely liquefied.

## Discussion

These results demonstrate for the first time that the US isolates of *P*. *hermaphrodita*, *P*. *californica*, and *P*. *papillosa* are lethal to *T*. *pisana*; and their lethal effects are comparable with iron phosphate at the higher recommended dose of 4.88 kg/m^2^ (1 lb/ft^2^). Although initially iron phosphate had a quick-kill effect on the snails, its overall effect was slightly less (87.8%) than any of the three *Phasmarhabditis* species, which caused 99–100% mortality at 4 DAE.

These results agree with the previous findings [37] on the lethal effects of naturally-occurring *P*. *hermaphrodita* on *T*. *pisana* in France. In a previously published laboratory assay, Coupland [37] found that the two isolates of *P*. *hermaphrodita* from *T*. *pisana* and *Trochoidea elegans* Gmelin, 1791 showed a similar level of virulence to *T*. *pisana* if applied on snails of <6 mm or >10 mm, with smaller snails dying faster than the larger-sized ones. In our assays, we used the size range (4–6 mm) similar to the small-sized snails (<6 mm), but a higher dose (5x the recommended rate of 30 DJs/cm^2^ vs 6/cm^2^) for all three *Phasmarhabditis* species, where mortality was noted 1 DAE and 99–100% was attained by 4 DAE. Differences in mortality and virulence level could be due to a variety of factors including genetic variation in isolates, bacterial associates of the nematode, snail immunity, and growth conditions associated with the laboratory set up. Additional assays are needed to determine the mortality at lower rates of application, e.g. the Nemaslug ® recommended rate of 30 DJs /cm^2^ and with larger snails exposed to these isolates of *Phasmarhabditis*. Previous studies of gastropod mortality caused by *Phasmarhabditis* spp. suggests that mortality can be rate-, size-, or age- dependent [33, 40]. Depending on results, the application rate may be lowered to Nemaslug® recommendation of 30 DJs/cm^2^.

Typically, *T*. *pisana* climb and aestivate on tall structures or plants from spring to summer [41] and remain attached on branches or leaves. This behaviour decreases the likelihood of *Phasmarhabditis* encountering *T*. *pisana* during certain seasons, since the nematodes are not likely to climb up plants in the absence of a thin film of water to move and survive. It has been suggested that baits are an effective control method prior to egg-laying from fall to spring [42, 43]. *T*. *pisana* eggs are laid on moist soil, and during late spring young snails start to climb vertical surfaces. Therefore, when the weather is cooler, and neonates are on the soil feeding on decaying matter, the nematodes could be applied directly on the soil by sprayer or hand sprinkler. This method may also be combined with manual picking of those snails that survive or are already mature. Our results suggest that any of the three US *Phasmarhabditis* species and isolates tested here could be valuable in mitigating *T*. *pisana* infestations, targeting younger snails prior to aestivation. Given additional information on the nematodes’ efficiency at lower doses, proper timing of application, impact on non-target organisms, among others; this approach could be considered an important tool for *T*. *pisana* IPM in California.

All three species of *Phasmarhabditis* used in this report were isolated from slugs recovered from nurseries and garden centers located in the state of California. In the case of *P*. *hermaphrodita* (Nemaslug ®), studies showed it does not cause mortality to some non-target organisms like *Ariolimax columbianus* Gould, 1851; *Pomatias elegans* Müller, 1774; *Monacha cantiana* Montagu, 1803; *Eisenia fetida* Savigny,1826; and *Lumbricus terrestris* Linnaeus, 1758 [44–47].

## Conclusion

Our laboratory assays have demonstrated that the US isolates of *P*. *hermaphrodita*, *P*. *californica* and *P*. *papillosa* at 150 DJs/cm^2^, caused significant mortality and are equally lethal to *T*. *pisana*. Their molluscicidal effects are comparable with iron phosphate at the recommended high dose of 4.88 kg/m^2^. The use of any of these gastropod-killing nematode species in California may provide a safe and environmentally friendly strategy for mitigating the potential damage and further spread by these snails, which are currently limited to San Diego and Los Angeles counties. However, additional trials are needed to determine their efficacy at the recommended rate of 30 DJs/cm^2^ for Nemaslug®, and whether the molluscicidal effects of the nematodes are dependent on the size or age of the snails.

## Supporting information

S1 Data File The data included here are the accumulated results of 3 replicates; 10 snails per arena, 3 arenas per replicate. The top section has the proportion of snails that died in each experiment. The bottom set shows the maximum, minimum, range, mean, standard deviation, and standard error of the mean for each replicate.
(XLSX)
